# First complete mitochondrial genome data from ancient South American camelids - The mystery of the *chilihueques* from Isla Mocha (Chile)

**DOI:** 10.1038/srep38708

**Published:** 2016-12-08

**Authors:** Michael Westbury, Stefan Prost, Andrea Seelenfreund, José-Miguel Ramírez, Elizabeth A. Matisoo-Smith, Michael Knapp

**Affiliations:** 1 Department of Mathematics and Natural Sciences, Evolutionary and Adaptive Genomics, Institute for Biochemistry and Biology, University of Potsdam, Karl-Liebknecht-Str. 24-25, 14476 Potsdam, Germany; 2 Department of Integrative Biology, University of California Berkeley, 3040 Valley Life Sciences Building, Berkeley, CA 94720-3140, USA; 3 Department of Biology, Stanford University, 371 Serra Street, Palo Alto, CA 94305-5020, USA; 4 School of Anthropology, Faculty of Social Sciences, Universidad Academia de Humanismo Cristiano, Condell 506, Santiago 7500828, Chile; 5 Centro de Estudios Avanzados, Universidad de Playa Ancha, Traslaviña 450, Viña del Mar, Chile; 6 Department of Anatomy, University of Otago, 270 Great King Street, Dunedin 9016, New Zealand

## Abstract

In South American societies, domesticated camelids were of great cultural importance and subject to trade and translocation. South American camelids were even found on remote and hard to reach islands, emphasizing their importance to historic and pre-historic South American populations. Isla Mocha, a volcanic island 35 km offshore of Central-South Chile, is an example of such an island. When Dutch and Spanish explorers reached the island in the early 17th century, they found that domesticated camelids called “*chilihueque*” played a major role in the island’s society. The origin and taxonomy of these enigmatic camelids is unclear and controversial. This study aims to resolve this controversy through genetic analyses of Isla Mocha camelid remains dating from pre-Columbian to early historic times. A recent archaeological excavation of site P21-3 on Isla Mocha yielded a number of camelid remains. Three complete mitochondrial genomes were successfully recovered and analysed. Phylogenetic analyses suggest that “*chilihueque*” was a local term for a domesticated guanaco. Results from phylogeographic analyses are consistent with Isla Mocha camelids being sourced from Southern Chilean guanaco populations. Our data highlights the capability of ancient DNA to answer questions about extinct populations which includes species identity, potential translocation events and origins of founding individuals.

The translocation of animals to islands has been an important human adaptive strategy even before the development of domestication^1^. Live animals are transported for numerous reasons including as future food resources, for their by-products, which can include bones, teeth, fibre, or dung, or for their cultural/spiritual or ritual importance. Often sizable challenges and obstacles had to be overcome to transport larger animals across considerable water gaps, emphasizing the cultural importance of these translocated animals. The enigmatic camelids of Isla Mocha, an island located approximately 35 km off the coast in the Arauco region of South Central Chile, are an example of such a challenging translocation of animals.

Isla Mocha has not been connected to the mainland since it rose above sea level during the Pleistocene, and it has no native large land mammals^2^,^3^. The earliest human occupation of the island is dated to about 3500 years ago and evidence of permanent occupation dates from about 1500 years ago^4^,^5^. Archaeological excavations on Isla Mocha have provided a large number of bone artifacts made of large mammal bones such as cetaceans, pinnipeds and camelids. Camelid bones make up the majority of the mammal remains in archaeological sites on the island. The large numbers of camelid remains present in archaeological sites on Isla Mocha suggest animals were likely living there from the time of permanent occupation. It is generally accepted that they were culturally important, used for ritual sacrifice, with the meat only consumed on special occasions^2^. Thus, live animals had to be translocated from the mainland to the island. The 35 km crossing from the mainland to Isla Mocha is a dangerous one, particularly in the winter time. Modern kayakers claim that the crossing from Tirua, the closest launching site on the mainland, to the island takes approximately 7 hours^2^. Transporting live camelids to Isla Mocha would have been particularly difficult and thus may have been a rare occurrence.

The origin and taxonomy of the Mocha camelids has been a matter of debate^2^. Early European descriptions of the island’s resources, including that of van Speilbergen in 1615, describe the presence of sheep or odd looking camel like creatures: “sheep of a very wonderful shape, having a very long neck and a hump like a camel, a hare lip and very long legs”^6^. These animals were referred to as “*chilihueque*”^6^.

Today, South American camelids are classified as belonging to four species within two genera, the wild forms, guanaco (*Lama guanicoe*) and vicuña (*Vicugna vicugna*), and the domestic species, llama (*L. glama*), and alpaca (*V. pacos*)^7^. How “*chilihueques*” fit into this taxonomy is unclear. Bonacic (1991) described them as a group of camelids that existed during Pre-Hispanic and early colonial times in central and south-central Chile, which went extinct during the 16th or 17th century, likely due to the arrival of the European horse and sheep^8^. The author suggested that “*chilihueque”* was either a local name for a different form of the llama that was brought down from Northern Chile or that “chilihueques” were a sub-species of locally domesticated guanaco^8^. Gay^9^, on the other hand, referred to “chilihueque” simply as the Mapuche name for a domesticated guanaco without describing them as a separate taxonomic unit.

Here we use next generation sequencing technology coupled with DNA bait capture enrichment to obtain complete mitochondrial genomes of camelid remains recovered from archaeological excavations on Isla Mocha in order to identify the camelid species translocated to Isla Mocha and locate the likely origin of the potential source population.

## Results

### DNA preservation, recovery and authenticity

A total of 14 samples were excavated and DNA was extracted from all samples. Ion Torrent sequencing libraries were prepared from all sample DNA extracts alongside three extraction blanks and a library preparation blank. All libraries including blank libraries were quantified using quantitative PCR with Ion Torrent adapter specific primers and checked for the presence of adapter dimers on an agarose gel. Of the 14 excavated samples from Isla Mocha, six samples yielded sequenceable libraries free of adapter dimers and of those, three libraries successfully recovered nearly complete camelid mitochondrial genome data (Table 1). All blanks produced alongside the successfully sequenced samples Mocha04, Mocha05, Mocha06 showed no detectable PCR products when amplified with a camelid specific primer test set targeting a 60 bp fragment of the camelid mitochondrial control region.

To further evaluate the authenticity of the obtained DNA sequence data, we inspected sequencing reads for characteristic deamination damage. The analysis with mapDamage showed damage patterns indicative of ancient DNA with the presence of increased C–T and G–A mutations at the respective 5′ and 3′ ends of sequenced molecules (see Supplementary Fig. S1). The average fragment length was 85–100 bp and therefore rather short, also consistent with ancient DNA (see Supplementary Fig. S2).

Ion Torrent sequencing reads from each sequencing library were mapped against both a *L. guanicoe* and a *V. vicugna* reference mitochondrial genome to identify potential ascertainment bias. In each case a higher coverage was achieved when mapping against *L. guanicoe* but both consensus sequences from the three samples respectively were identical independent of whether the sequencing reads were mapped against guanaco or vicuña. Phylogenetic analyses were conducted with the consensus sequences mapped to the guanaco due to the higher coverage mappings.

### Phylogenetic and phylogeographic analyses

**Figure 1:**
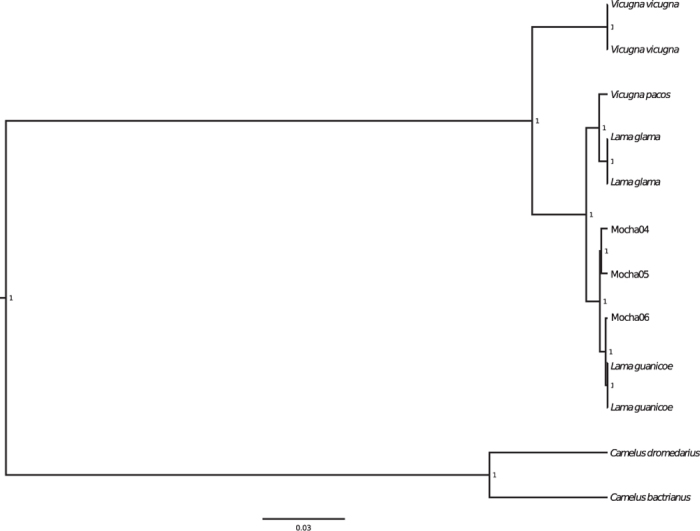
Bayesian reconstruction of phylogenetic relationships of Isla Mocha camelids. Tree reconstructed with complete mitochondrial genome data from all extant South American camelids with two *Camelus* species as outgroup. Numbers at the branch nodes indicate posterior probability support values. All three Mocha samples are shown to group together with the *Lama guanicoe* mitogenome sequences with high support values.

Bayesian phylogenetic reconstructions yielded a well-resolved tree topology with high posterior probabilities on all branches. Complete mitochondrial genome sequences of all three sequenced camelid samples from Isla Mocha grouped unambiguously with *L. guanicoe* (Fig. 1).

**Figure 2:**
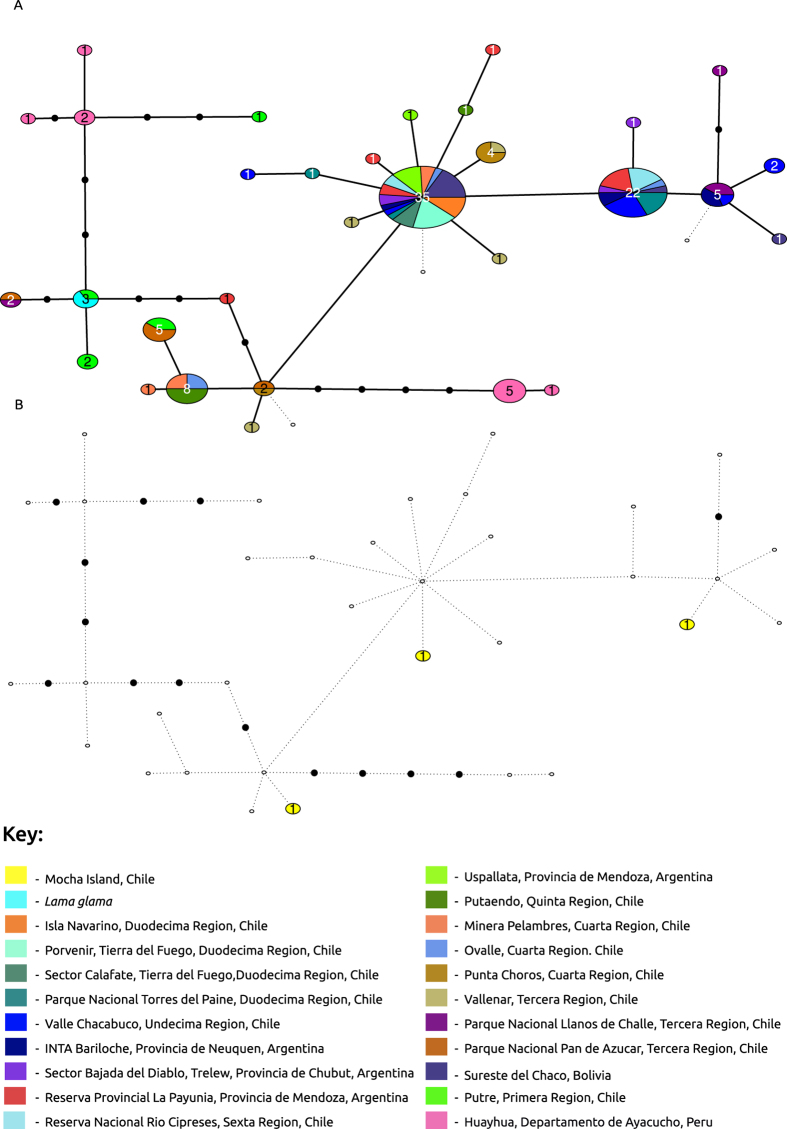
Haplotype network constructed using mitochondrial control regions of extant guanaco, llama and Isla Mocha camelids. Each node represents a single mutation; colours correspond to regions of origin. (**A**) Present day camelids sequenced by Marín *et al*.^10^. (**B**) Ancient Isla Mocha camelids from this study in relation to Marín *et al*.^10^ camelids. Isla Mocha samples are shown to fall into three separate parts of the network while all being distinct from any living guanaco or llama control region sequence.

Phylogeographic analyses were conducted on control region data only, with the 176 guanaco control regions published by Marin *et al*.^10^ being used as reference data. All three Isla Mocha camelids had different control region haplotypes, and all Mocha haplotypes differed from the published reference data (Fig. 2).

Mocha04 was most closely related to and one mutation removed from a haplotype shared between guanaco found in 2 different regions of Southern South America: in Punta Choros of the Cuarta Region (now Coquimbo region), Chile and Parque Nacional Pan de Azúcar of the Tercera Region (now Atacama Region), Chile.

Isla Mocha sample, Mocha05, was just one substitution removed from a common and widespread control region haplotype that is shared between 13 different guanaco populations from 13 different areas of South America. These populations were those from “Sureste del Chaco Boliviano, Bolivia”, “Sector Bajada del Diablo, Trelew, Provincia de Chubut, Argentina”, “Minera Pelambres, Cuarta Región, Chile” (now Coquimbo region), “INTA Bariloche, Provincia de Neuquén, Argentina”, “Reserva Provincial La Payunia, Provincia de Mendoza, Argentina”, “Valle Chacabuco, Undécima Región (now Aysén Region), Chile”, “Reserva Nacional Río Cipreses, Sexta Región (now General L. Bernardo O’Higgins Region), Chile”, “Uspallata, Provincia de Mendoza, Argentina”, “Ovalle, Cuarta Región. Chile” (now Coquimbo region). “Isla Navarino, Duodécima Región, Chile” (now Magallanes and Chilean Antartica Region). Porvenir, Tierra del Fuego, Duodécima Región, Chile” (now Magallanes and Chilean Antartica Region). “Sector Calafate, Tierra del Fuego, Duodécima Región, Chile” and “Parque Nacional Torres del Paine, Duodécima Región, Chile” (now Magallanes and Chilean Antartica Region).

Mocha06 was one substitution away from three different regional populations. These regional populations coming from “Valle Chacabuco, Undécima Región, Chile” (now Aysén Region), “INTA Bariloche, Provincia de Neuquén, Argentina”, “Parque Nacional Llanos de Challe, Tercera Región (now Atacama Region), Chile”.

**Figure 3:**
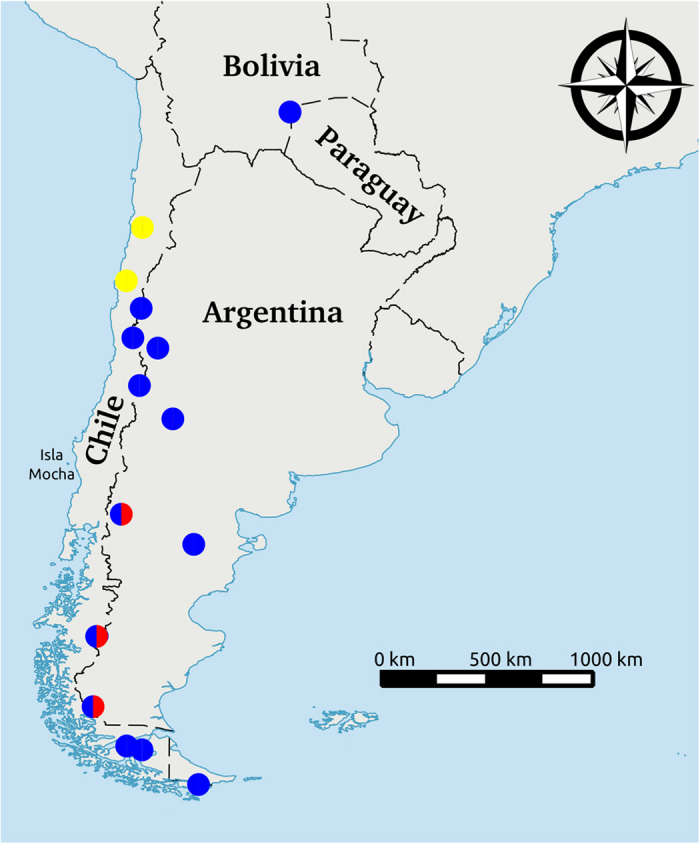
Map of the genetically closest guanaco haplotype to our Isla Mocha camelids’ sequences. The geographic location of the genetically closest guanaco haplotype to Mocha04 is indicated in yellow, to Mocha05 is indicated in blue and to Mocha06 is indicated in red. Map generated using QGIS 2.0.1-Dufour (QGIS Development Team, 2016. QGIS Geographic Information System. Open Source Geospatial Foundation Project. http://www.qgis.org/).

All the above populations that are closely related to the Mocha camelids, with the exception of “Sureste del Chaco Boliviano”, are found south of 26’S (approximately the latitude of the border between Argentina and Bolivia) (Fig. 3). All guanaco populations from north of 20’S sequenced by Marin *et al*.^10^ are more distantly related to the Mocha camelids and at least three mutations removed from any of the Mocha samples.

## Discussion

Our study helps to reconstruct the identity of the enigmatic camelids from Isla Mocha. Bayesian phylogenetic analyses, using the complete mitochondrial genomes of three ancient camelids grouped these individuals with modern *L. guanicoe*, to the exclusion of *Vicugna vicugna, Lama glama* and *Vicugna pacos* (Fig. 1). These results are consistent with previous analyses based on morphological data, which also identified the Isla Mocha camelids as guanacos^2^.

Identifying the exact geographic location of the source population of the Isla Mocha guanacos is challenging. Very limited complete mitochondrial data from guanaco populations from across South America is available to date. The only large-scale phylogeographic information for guanacos was based on the mitochondrial control region only^10^. Marín *et al*. discovered limited phylogeographic structure of guanacos across their range, but did find a well-defined separation between northern populations from Peru, Bolivia and Northern Chile and southern populations from Argentina and southern Chile. The only exception from this structure is one isolated population in the highlands of Bolivia, which groups with the southern haplotypes. The three Isla Mocha individuals sequenced in this study had unique and previously unsequenced haplotypes, but did group most closely with the southern populations.

These results suggest that the Isla Mocha camelids were most likely derived from southern South American guanaco populations rather than llamas brought in from the North, as suggested by the alternative hypothesis. The very limited phylogeographic structure of southern South American guanacos prevents a more precise determination of the geographic origins of the Isla Mocha camelids.

Together with morphological studies, our data provides some insights into the taxonomic status of the enigmatic “*chilihueques”* from Isla Mocha. All pre-historic and early historic camelid remains from Isla Mocha studied so far have been described as guanacos based on morphological evidence^2^,^4^,^11^. Similarly, our genetic data group the three individuals studied by us with guanacos. While the haplotypes of our samples are unique, they do not form a monophyletic clade within the guanaco diversity as could for example be expected from a reproductively isolated subspecies. We therefore suggest that “*chilihueque”* was merely a local term for a guanaco. Whether or not the term “*chilihueque”* was specifically referring to domesticated guanacos, as suggested by Gay^9^, cannot be inferred from our limited genetic data or the island’s archaeology and remains a contentious question^2^. It is conceivable that the animals were transported to the island and released for hunting or that they were casually managed rather than fully domesticated.

Our data provide the first glimpse into the origin and taxonomy of the enigmatic Isla Mocha camelids or “c*hilihueques”*, reinforcing the hypothesis that these camelids were guanacos derived from local mainland populations rather than domesticated llama populations brought in from further north. This result suggests a local sourcing of animals for translocation to the isolated Isla Mocha.

## Methods

In 2011, an archaeological excavation was undertaken on Mocha at site P21–3 located on Parcela 21 on the southwest site of the island. The site is located on a marine terrace approximately 20–30 m above sea level. Two units, 100 and 100a were identified. A total of 10 m^3^ was excavated in unit 100a and total of 6.4 m^3^ in unit 100. Excavations followed the natural stratigraphy of the sites. An area of 2 × 4 metres was excavated to a depth of 2 meters, in approximately 10 cm spits. P21-3 is believed to be an occupation site and midden linked to P21-1, a burial site located approximately 20 metres away and excavated in 2001 and 2003 by Daniel Quiroz and colleagues^12^. The P21-3 site contains evidence of continuous human occupation for at least 1,000 years. A number of intact cultural deposits containing a large amount of faunal, ceramic and lithic material from throughout the El Vergel complex (dating to 1,000–1,500 AD) and into the Pitren occupation period (0–600 AD) provide evidence for occupation over this time. Faunal remains recovered included fish, marine mammals, camelids, rodents, canids, and bird bones (unpublished data). The sequenced camelid remains were recovered from archaeological contexts at P21-3, with associated material dating from 689 +/− 31 BP to 134 +/− 20 BP (Wk 38379–38395). A total of 14 camelid samples were considered appropriate for ancient DNA analysis and were bagged and labeled for such purposes at the site.

### DNA extractions

All ancient DNA extractions and sequencing library preparations were undertaken in a dedicated ancient DNA laboratory, at the University of Otago, Dunedin. 250 mg of bone material per sample was ground to a powder using a clean mortar and pestle then subsequently extracted using a silica based extraction protocol modified from Rohland and Hofreiter^13^. For every five samples extracted, one extraction blank was also processed under the same conditions. Three microliter aliquots of extraction blanks were amplified with a primer set (Supplementary Table S1), based on sequences from Marín *et al*.^10^, targeting a 60 bp fragment of the camelid mitochondrial control region to detect the presence of camelid DNA in extractions and blanks. The following thermal profile was used for this amplification: Initial denaturation step of 94 °C for nine minutes followed by 40 steps of 94 °C denaturation for 20 seconds, 55 °C annealing for 30 seconds, and 72 °C elongation for 30 seconds then a final elongation of 72 °C for 4 minutes and storage at 4 °C. PCR products were visually checked for amplification bands via agarose gel electrophoresis on a 2.5% agarose gel.

### Ion torrent library preparation

Barcoded Ion torrent libraries were produced from all DNA extracts, extraction blanks and no-template controls by ligating ion torrent specific sequencing adapters to the ancient DNA extracts using a modified version of the ancient DNA library build protocol described by Knapp *et al*.^14^. Subsequent to library preparation, barcoded libraries were PCR amplified to produce easy to store stock libraries while at the same time retaining maximum library complexity^14^. To reduce amplification artifacts, the PCR was stopped after amplification plateau was reached, as determined by quantitative PCR.

Agarose gel electrophoresis (2.5% gel) of amplified libraries was used to visually check the success of the library preparation and to identify the presence of adapter dimers. Libraries with strong adapter dimers were excluded from sequencing as they strongly reduce the reads on target in a sequencing run. Only libraries that showed a characteristic smear of expected size (c. 150–300 bp) and no adapter dimer bands were used for downstream analyses. To check for potential cross contamination, no-template control libraries were PCR amplified with the same 60 bp camelid control region specific primers and the same thermal profile used to test for contamination of the extraction blanks (Supplementary Table S1). Three sequencing libraries passed all quality checks and were used for downstream analyses. Amplified libraries were purified using a Qiagen MinElute kit following the manufacturers’ protocol.

### Hybridisation capture

Sequencing libraries were enriched for mitochondrial DNA using hybridisation capture enrichment^15^. Two micrograms of each library were required for hybridisation capture. To obtain this amount of template library, four 1 μl aliquots of each stock library were PCR amplified in 100 μl reaction volume each with reactions again being terminated after reaching PCR amplification plateau. The four amplicons from each library were then pooled, purified using a Qiagen Minelute kit, and eluted in 20 μl of 0.1xTE. DNA extracted from modern *Vicugna pacos* (alpaca) samples was used to produce the capture bait as described in Maricic *et al*.^15^. In brief, modern samples were extracted using a Qiagen DNeasy extraction kit, complete mitochondrial genomes were amplified by long range PCR as two separate fragments using primers based on the *Vicugna pacos* mitochondrial genome NCBI Reference Sequence: NC_002504.1 (Supplementary Table S2), purified using Qiagen QIAquick DNA purification kit and quantified using a thermo fisher scientific nanodrop 2000c spectrophotometer. Long range PCR products were pooled in 750ng equimolar amounts and fragmented through sonication. Biotinylated adapters were ligated to the sonicated mitochondrial genome fragments. Adapter ligated fragments were then bound to streptavidin coated beads to be used as bait in the hybridisation capture.

### Sequencing

Capture enriched, barcoded libraries were pooled in equimolar ratios, depending on quantities determined by qPCR, and sequenced on an Ion Torrent sequencer at the University of Otago, Dunedin.

### Raw data processing and mitochondrial genome mapping

Ion torrent sequencing reads were separated according to their barcode and processed following Clarke *et al*.^16^. Any adapter sequences and reads shorter than 25 bp were trimmed using cutadapt^17^. Processed reads were mapped against the guanaco and vicuña reference mitochondrial genomes NC_011822.1 (guanaco) and FJ456892.1 (vicuña) using the Burrows-Wheeler Aligner (BWA)^18^. Duplicate read removal and quality control were performed using SAMtools (a tool for interacting and post-processing short DNA read alignments) while consensus sequences (bcftools and mpileup) and coverage plots were constructed using SAMtools mpileup^19^. Consensus sequences were submitted to GenBank (accession numbers: KX388532 - KX388534, see Table 1). The perl script make_coverage_plots.pl was used to visualize the coverage plots (see Supplementary Fig. S3).

The commonly used Python and R computational framework known as mapDamage was implemented to track and quantify ancient DNA damage patterns in order to assess for ancient DNA authenticity^20^.

### Phylogenetic analysis

A multiple sequence alignment (MSA) was performed using the three consensus Mocha camelid mitochondrial genomes alongside guanaco, llama, alpaca, vicuña and outgroup sequences obtained from NCBI Genbank (Supplementary Table S3) using Mafft with parameters specified for accuracy over speed^21^. The MSA was inspected by eye using Jalview^22^, all sites with an unknown base (n) were removed. BEAUti was used to prepare alignments for phylogenetic tree reconstruction, which was carried out using BEASTv1.8.1. Parameters included a HKY+G substitution as determined using jmodeltest2.1.7^23^ and a Speciation:Birth-Death process tree prior^24^. The Markov Chain Monte Carlo (MCMC) was run from a random starting tree for 10,000,000 iterations, sampling every 1,000th tree with a burn in of 100,000 states. Effective sample size for estimated parameters was checked using Tracer v. 1.6^25^. All parameters were estimated from at least 200 fully independent trees sampled by the MCMC. The MCMC was run three times and results were compared to check for convergence of the algorithm. Posterior probabilities were annotated onto the BEAST output tree using TreeAnnotator.

### Network analysis

As phylogenetic analyses indicated that Isla Mocha camelids were guanacos, a network analysis against a guanaco dataset was undertaken in order to fit the Isla Mocha camelids into the phylogeographic framework of wild guanacos and identify their potential region of origin. Due to lack of sufficient complete mitochondrial genomes available for comparison, the dataset was limited to the control region of the mitochondrial genome, for which an extensive phylogeographic reference database is available^10^. These control regions were aligned with the Isla Mocha camelid samples using Mafft, then analysis and network construction was performed using the freely available R script, TempNet^26^.

## Additional Information

**How to cite this article**: Westbury, M. *et al*. First complete mitochondrial genome data from ancient South American camelids - The mystery of the *chilihueques* from Isla Mocha (Chile). *Sci. Rep.* **6**, 38708; doi: 10.1038/srep38708 (2016).

**Publisher's note:** Springer Nature remains neutral with regard to jurisdictional claims in published maps and institutional affiliations.

## Supplementary Material

Supplementary Information

## Figures and Tables

**Table 1:** Excavation site and sequencing details for each successfully sequenced sample from Isla Mocha.

Sample	Site	Unit	Level	Average coverage per base site	Number of mapping reads	Percent of genome covered	GenBank reference number
Mocha04	P21-3	100	A	85.80x	12549	98.64%	KX388532
Mocha05	P21-3	100	150–160 cm	3.01x	528	89.56%	KX388533
Mocha06	P21-3	100a	190–200 cm	28.50x	3970	98.43%	KX388534
