# Supplementary material for: First complete mitochondrial genome data from ancient South American camelids - The mystery of the chilihueques from Isla Mocha (Chile)
Source: Sci Rep. 2016 Dec 8;6:38708. doi: 10.1038/srep38708 (PMC5144146; doi:10.1038/srep38708)
Supplement: Supplementary Information [file srep38708-s1.pdf]

Supplementary Information

First complete mitochondrial genome data from ancient South American camelids - The mystery of the *chilihueques* from Isla Mocha (Chile)

(SREP-16-28571)

Michael Westbury[1], Stefan Prost[2,3], Andrea Seelenfreund[4], José-Miguel Ramírez[5], Elizabeth A. Matisoo-Smith[6], Michael Knapp[6*]

[1] Department of Mathematics and Natural Sciences, Evolutionary and Adaptive Genomics, Institute for Biochemistry and Biology, University of Potsdam, Karl-Liebknecht-Str. 24-25, 14476 Potsdam, Germany

[2] Department of Integrative Biology, University of California Berkeley, 3040 Valley Life Sciences Building, Berkeley, CA 94720-3140, USA

[3] Department of Biology, Stanford University, 371 Serra Street, Palo Alto, CA 94305-5020, USA

[4] School of Anthropology, Faculty of Social Sciences, Universidad Academia de Humanismo Cristiano, Santiago 7500828, Chile

[5] Centro de Estudios Avanzados, Universidad de Playa Ancha, Traslaviña 450, Viña del Mar, Chile.

[6] Department of Anatomy, University of Otago, 270 Great King Street, Dunedin 9016, New Zealand

*Corresponding author: michael.knapp@otago.ac.nz

Supplementary Figure S1: Damage patterns indicative of ancient DNA constructed using mapDamage. A-G transitions indicated in blue and C-T transitions are indicated in red. A: Mocha04 B: Mocha05, C: Mocha06.

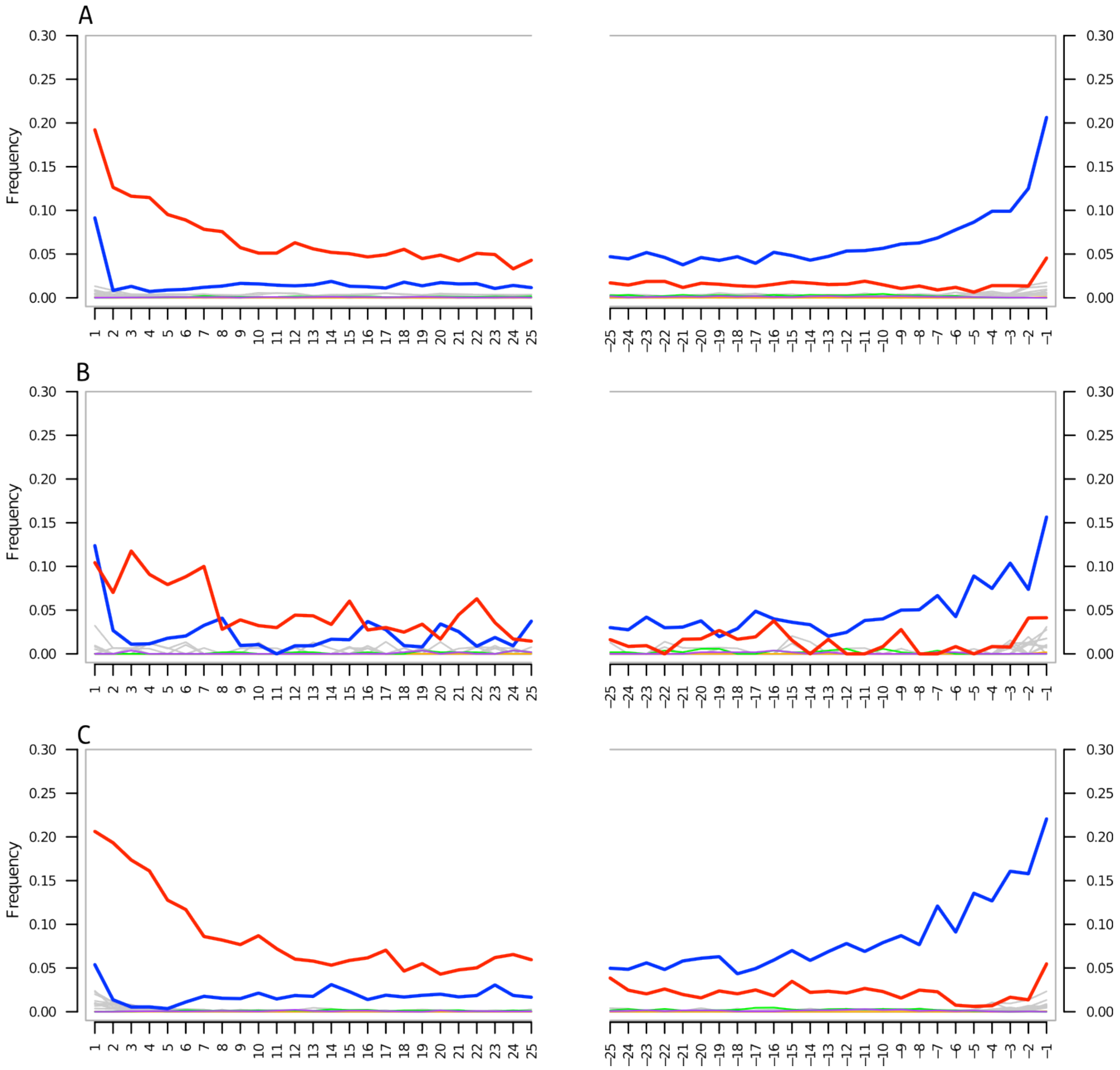

Supplementary Figure S2: Mapping fragment length patterns indicative of ancient DNA constructed using mapDamage. A: Mocha04 B: Mocha05, C: Mocha06.

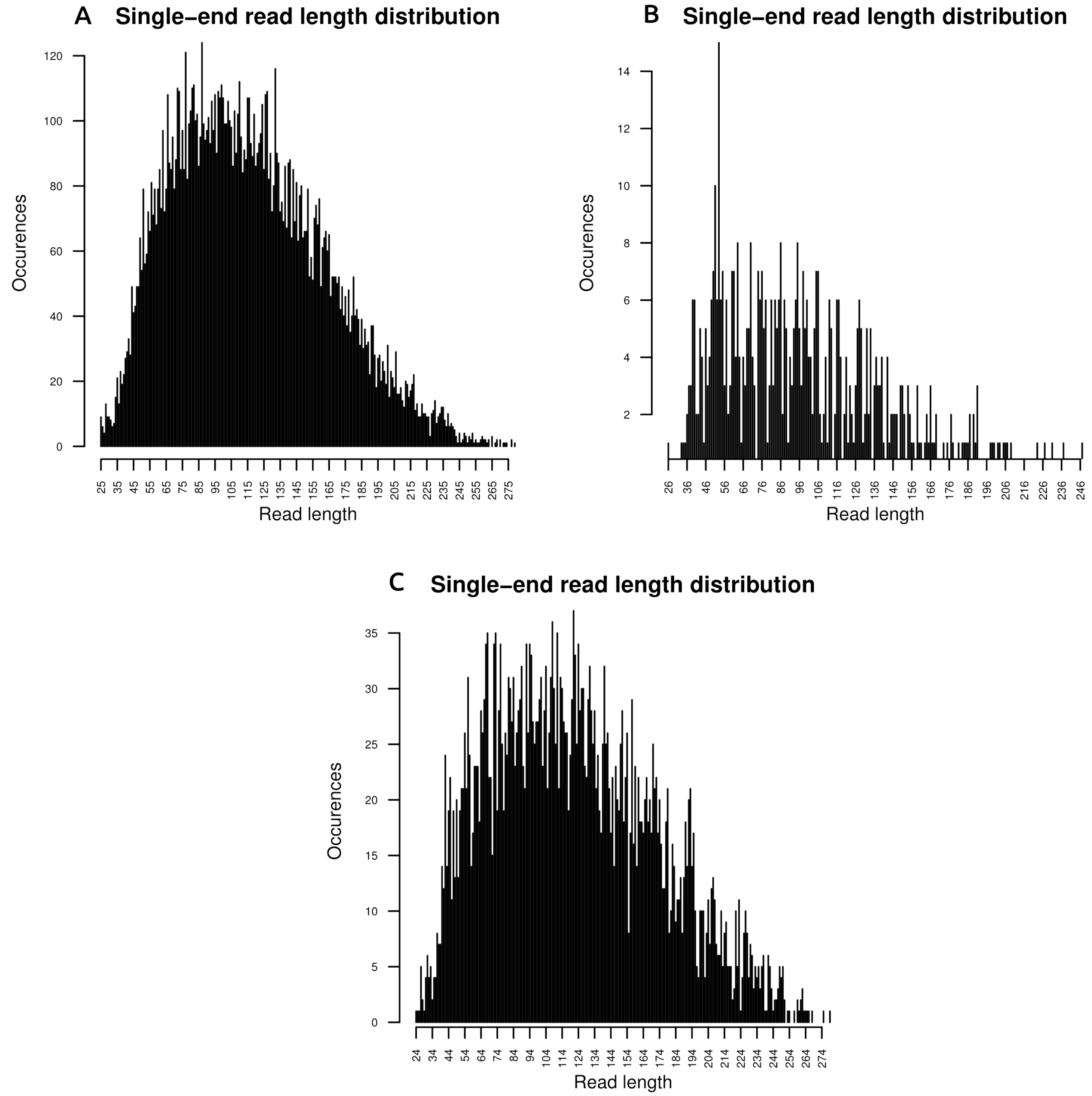

Supplementary Figure S3: Coverage plots of complete mitochondrial genomes with number of bases and percentage of the whole genome covered. Average coverage is indicated by the dotted line.

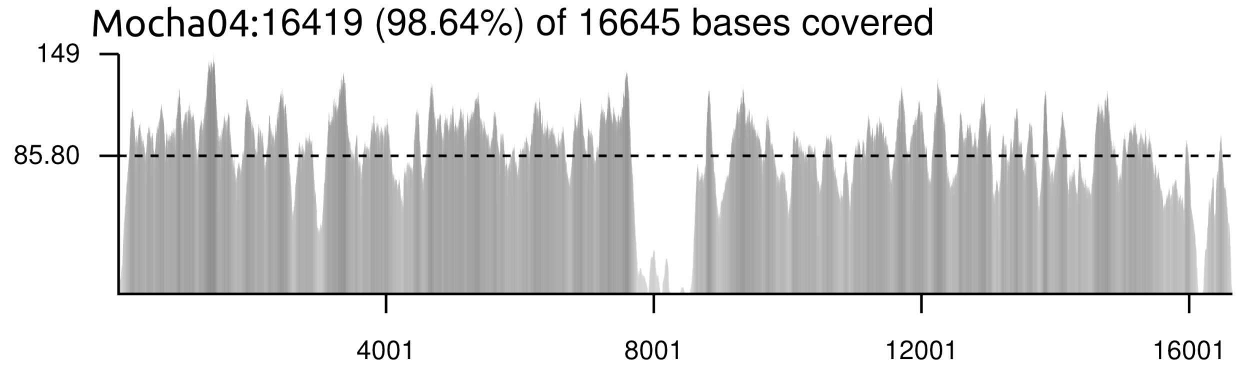

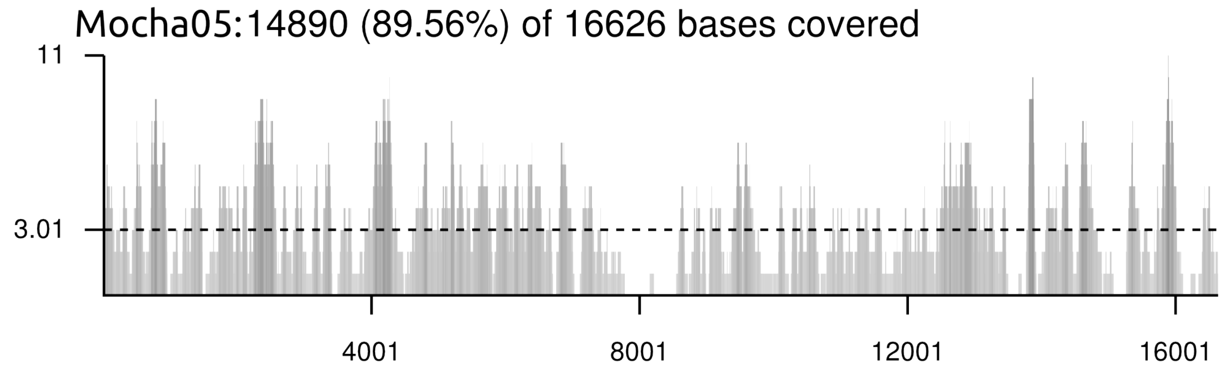

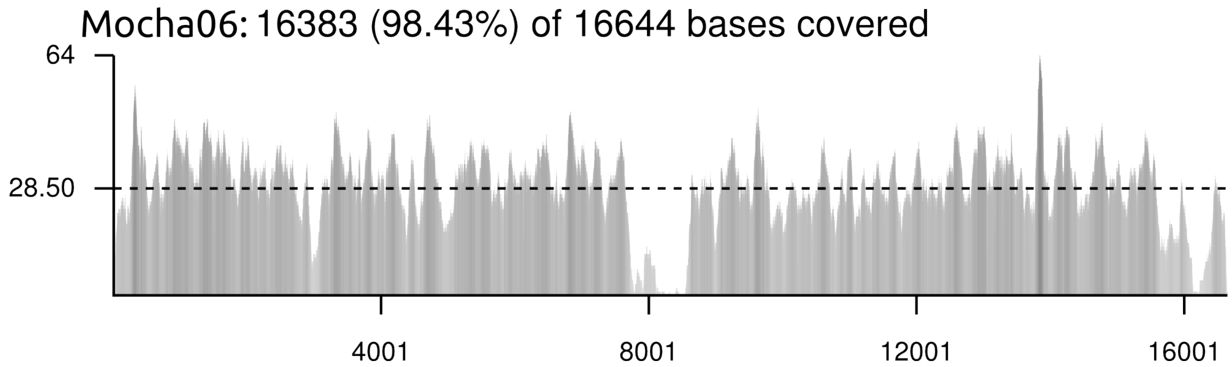

Supplementary Table S1: Primer sequences for the 60bp mitochondrial control region contamination test fragment

Primer names	Sequence 5'-3'
Cam_F	ACCACAGTTATGTGTGAGCATGGGC
Cam_R	CCAGGCATCTGGTTCTTACTTCAGGA

Supplementary Table S2: Long range primers used to amplify modern alpaca complete mitochondrial genome

Primer name	Primer sequence 5'- 3'
CamLR1_F	GGTTTGATCCCAGCCTTTCT
CamLR1_R	GCTCCTCCAATTAGGTGCAT
CamLR2_F	GGCCGTTCGACTAACAGCTA
CamLR2_R	CTTAGGGGGCATTCTCACTG

Supplementary Table S3: Species and NCBI genbank accession numbers for sequences used in MSA and whole mitochondrial genome phylogenetic analyses

Accesion number	Genus	Species
NC_009628.2	*Camelus*	*bactrianus*
NC_009849.1	*Camelus*	*dromedarius*
EU681954.1	*Lama*	*guanicoe*
FJ456892.1	*Vicugna*	*vicugna*
NC_011822.1	*Lama*	*guanicoe*
AP003426.1	*Lama*	*glama*
NC_012102.1	*Lama*	*glama*
NC_013558.1	*Vicugna*	*vicugna*
AJ566364.1	*Lama/Vicugna*	*pacos*
